# Metallic Ion Release Behaviors from Cobalt–Chromium Alloys Fabricated by Additive Manufacturing with Mechanical Grinding in an Acidic Saline Solution

**DOI:** 10.3390/ma18020432

**Published:** 2025-01-17

**Authors:** Naoto Sakurai, Tomofumi Sawada, Yukinori Kuwajima, Kenta Yamanaka, Naoyuki Nomura, Masaaki Kasahara, Akihiko Chiba, Kazuro Satoh, Shinji Takemoto

**Affiliations:** 1 Division of Orthodontic, Department of Developmental Oral Health Science, School of Dentistry, Iwate Medical University, Iwate 020-8505, Japan; nsakurai@iwate-med.ac.jp (N.S.); ykuwaji@iwate-med.ac.jp (Y.K.); kazsatoh@iwate-med.ac.jp (K.S.); 2 Department of Biomedical Engineering, Iwate Medical University, Iwate 028-3694, Japan; sawada@iwate-med.ac.jp; 3 Institute for Materials Research, Tohoku University, Sendai 980-8577, Japan; kenta.yamanaka.c5@tohoku.ac.jp; 4 Department of Materials Processing, Graduate School of Engineering, Tohoku University, Sendai 980-8579, Japan; naoyuki.nomura.a2@tohoku.ac.jp; 5 Department of Dental Materials Science, Tokyo Dental College, Tokyo 101-0061, Japan; kasaharamasaaki@tdc.ac.jp; 6 New Industry Creation Hatchery Center, Tohoku University, Sendai 980-8577, Japan; akihiko.chiba.d3@tohoku.ac.jp

**Keywords:** corrosion resistance, dental appliance, electron beam melting, selective laser melting, static immersion test

## Abstract

This study aimed to investigate the release of metallic ions from cobalt–chromium (Co-Cr) alloys fabricated by additive manufacturing (AM) for comparison with dental casting. Co-Cr alloys were fabricated via AM using selective laser melting (SLM) and electron beam melting (EBM) in powder-bed fusion. Polished and mechanically ground specimens were prepared. Each specimen was analyzed using an electron probe microanalyzer (EPMA). Each specimen was immersed in an acidic saline solution for 7 days in accordance with ISO 10271: 2020. The EPMA indicated the segregation of some elements in the as-prepared SLM and EBM specimens, whereas the polished and ground specimens exhibited a homogenous elemental distribution. The total amount of ion release from the SLM and EBM specimens was confirmed to be less than 7 μg/cm^2^, which was less than 42 μg/cm^2^ for the cast specimen. The polished and ground specimens exhibited an even lower ion release than the as-prepared specimens. The amount of ions released from the Co-Cr alloy was less than the 200 μg/cm² requirement of ISO 22674: 2022. Co-Cr alloys fabricated by SLM and EBM could provide superior corrosion resistance to cast specimens. AM could be a valuable method for fabricating appliances and denture frameworks in dentistry.

## 1. Introduction

Metallic materials are widely used in orthopedics, dental implants, crown restorations, and the frameworks of removable partial dentures because of their superior strength and resistance to fracture, although they do not natively constitute the body [1]. In the field of orthodontics, metals are used for various orthodontic devices, such as brackets, arch wires, and other removable and fixed prosthetic devices. Although orthodontic metal brackets (brackets) are less esthetic than those made from composite materials, polycarbonate, or ceramics, they are still widely used in clinical practice because of their low coefficient of friction between the wire and bracket, suitability for tooth movement, good mechanical properties, and low risk of wear and tear [2,3,4,5].

Brackets are mainly made from stainless steels, cobalt–chromium (Co-Cr) alloys, and titanium alloys, and are bonded to tooth surfaces using adhesives such as adhesive resin cement. The brackets bonded to the tooth surface are required to have not only the above-mentioned frictional resistance and mechanical properties, but also chemical durability in the oral environment. Some metallic materials can corrode the oral environment. The dissolution of metal ions due to corrosion may not only deteriorate the material but also cause allergic reactions. Therefore, it is necessary to select materials with excellent corrosion resistance in oral environments [6,7,8,9,10]. 

Brackets are produced by casting or injection molding. Computer-aided design (CAD) and computer-aided manufacturing (CAM) technologies for dental alloys have recently been applied to metallic restorations and denture frameworks. Subtractive manufacturing is a mainstream CAM; however, additive manufacturing (AM) technologies, which can produce complex shapes, have been applied clinically in recent years [11]. AM is broadly divided into seven types of built methods in ISO/ASTM 52900: 2021 [12]; among metal AM technologies, powder bed fusion (PBF) is primarily employed when AM is performed with metal materials. In PBF, a layer of metallic powder is melted and shaped using a laser beam (selective laser melting (SLM)) or an electron beam (electron beam melting (EBM)). Metallic products fabricated by AM have mechanical properties superior to those obtained by casting [13,14]. In addition, the mechanical properties of metal components, particularly their ductility, vary according to the direction of fabrication [13,14,15,16,17]. However, several challenges remain to be overcome, particularly the variations in the corrosion resistance of metallic orthodontic brackets, wires, and prosthetic devices in the oral environment, depending on the state of polishing and the surface conditions during the manufacturing process.

Metallic allergy is sometimes an issue for cobalt–chromium alloys due to the contents of chromium and nickel in their constituent elements. Generally, Co-Cr alloys were found to have superior corrosion resistance in several NaCl solutions with lactic acid [18,19,20] in the 1980s, and have very low cytotoxicity [21] due to the formation of a passive chromium oxide film. Grosgogeat et al. reported in a review that patients who react to cobalt or chromium may have allergic reactions, but the probability of this is extremely low compared to titanium alloys [22]. These in vitro tests were performed in various solutions, such as simulated body fluid, acidic saline solution, and culture medium, and several conditions (temperature and pH) [23]. Brackets and denture base frameworks are used in some parts in their as-cast or form without being ground or polished. However, polished specimens are commonly used in normal material testing, but the corrosion resistance of the specimens as they are formed has not been examined.

In this study, we aimed to evaluate the corrosion resistance of various Co-Cr alloys fabricated by SLM and EBM by comparing their release behavior with that of cast alloys in an acidic saline solution, in accordance with ISO 10271: 2020 [24]. The ISO 10271 provides corrosion test methods for metallic materials in dentistry, and the static immersion test in acidic saline solution was among the standard. To evaluate the additive manufacturing technique with grinding and/or polishing as an availability of dental appliance and prosthodontic devices was performed to be used the static immersion test for cobalt-chromium alloy fabricated by additive manufacturing. The null hypothesis was that the built, polished, and ground Co-Cr specimens would have similar metallic ion release compared to conventional cast specimens.

## 2. Materials and Methods

### 2.1. Specimen Preparation

Three types of Co-Cr specimens, two types of SLMs, and an EBM were fabricated by PBF-AM and compared with specimens produced by a conventional casting method. The SLMs and EBM specimens were fabricated flat without support from the bottom plate. Disc-shaped specimens with a 16 mm diameter and 2 mm thickness were fabricated by SLM using a hybrid metal 3D-build up and grinding machine (LUMEX Avance-25: Matsuura Machinery Corp., Fukui, Japan) with Co-Cr powder (Matsuura Cobalt chrome, Matsuura Machinery Corp.) (denoted as SLM1). The fabrication process utilized an Yb fiber laser with an energy density of approximately 100 J/mm^3^ and a spot diameter of 0.1 mm under a nitrogen gas atmosphere. 

A different Co-Cr alloy powder (Remanium Star, Dentaurum, Ispringen, Germany) was processed using another SLM machine (Concept Laser Mlab R, GE Additive, Lichtenfels, Germany) (denoted as SLM2). The fabrication parameters included an Yb fiber laser with an energy density of approximately 125 J/mm^3^ and a spot diameter of approximately 0.05 mm.

The third type of AM specimen was fabricated using EBM (Arcam A2X, GE Additive, Gothenburg, Sweden). The specimens were fabricated in a vacuum with dimensions of 15.5 mm × 15.5 mm × 35 mm, a current of 4.5–18 mA, and a scan speed of 210–440 mm/s. A wire-cut electric discharge machine (SL400G; Sodick, Kanagawa, Japan) was used to cut 2 mm thick specimens from each block. The final built-up surface was used as an as-built specimen (denoted as EBM). 

A disk-shaped resin pattern with a diameter of 16 mm and a thickness of 2 mm was fabricated using liquid bath photopolymerization. The pattern was embedded in a phosphate investment material (Snow White, Shofu Inc., Kyoto, Japan), which was heated according to the conventional method. Co-Cr ingots (Baltron, IDS Corp., Tokyo, Japan) were cast using a high-frequency induction casting machine (Cascom S, DENKEN-HIGHDENTAL Co., Ltd., Kyoto, Japan) (denoted as CAST).

In this study, we evaluated the corrosion behaviors of as-built (AS), mechanically ground (ME), and polished (PO) surfaces. One-third of the SLM1-AS specimens had their surfaces mechanically ground using a hybrid metal 3D-build up and grinding machine (LUMEX Avance-25: Matsuura Machinery Corp.,) (SLM1-ME). All specimens were embedded in epoxy resin (SCANDIPLEX, SCANDIA GmbH, Hagen, Germany), and one-third of the SLM1-AS, SLM2-AS, EBM-AS, and CAST-AS was polished with P1200 water-resistant abrasive paper (denoted as SLM1-PO, SLM2-PO, EBM-PO, and CAST-PO, respectively) (*n* = 6). All specimens were sonicated in distilled water for 10 min, and excess water was removed using filter paper. The metallic powder compositions and manufacturers of the four Co-Cr alloys used in this study are presented in Table 1.

### 2.2. Specimen Characterization

The crystal structure of the specimens was examined using a horizontal multi-purpose X-ray diffractometer (XRD: Ultima IV, Rigaku Corp., Tokyo, Japan); the X-ray source was a CuKα irradiation accelerating at 40 kV–40 mA with a scan speed of 1°/min and a 2θ range of 20° to 100°.

The elemental composition of the surface was analyzed using an electron probe microanalyzer (EPMA; JXA-8530F, JEOL Ltd., Tokyo, Japan). Specimen surfaces were observed using a scanning electron microscope (SEM; SU8010, Hitachi High-Tech Corp., Tokyo, Japan). Before the SEM and EPMA observations, the specimens were coated with OsO_4_ by an osmium coater (OPC60A, Filgen Inc., Aichi, Japan).

### 2.3. Static Immersion Test

The test solution was dissolved in 1 L distilled water to 5.85 g of sodium chloride (99.99% NaCl, FUJIFILM Wako Pure Chemical Corp., Osaka, Japan), and 10.0 g of lactic acid (KANTO Chemical Co., Inc., Tokyo, Japan) was added to prepare an acidic saline solution (pH 2.3 ± 0.1) according to ISO 10271: 2020 [24]. The specimen, as prepared in 2.1, was immersed in 10 mL of acidic saline solution per 100 mm^2^ in polystylen bottle and held in a thermostatic chamber set at 37 °C for 7 days.

After the immersion, the pH of the solution was measured using a pH meter (F-24; HORIBA Ltd., Kyoto, Japan). The concentration of metallic ions in the solution was measured using an inductively coupled plasma optical emission spectrometer (ICP; Vista-MPX, SII, Chiba, Japan) and the calibration curve method. The wavelengths of the elements used in the ICP measurements were Co, 231.160 nm; Cr, 267.716 nm; Fe, 259.940 nm; Mn, 257.610 nm; Mo, 281.615 nm; Ni, 231.604 nm; Si, 288.158 nm; and W, 220.449 nm. Calibration was performed at concentrations of 0.1, 0.5, and 1.0 mg/L for each element, and the wavelengths with a correlation coefficient of 0.995 or higher for that calibration curve were determined in the test solution.

### 2.4. Statistical Analysis

Statistical analysis was performed using Welch’s one-way analysis of variance (ANOVA), followed by Tukey’s multiple comparison test at a significance level of 5%. The total number of ion released between AS and PO specimens was determined using Student’s test at a significance level of 5%.

## 3. Results

### 3.1. Materials Characterization

**Figure 1:**
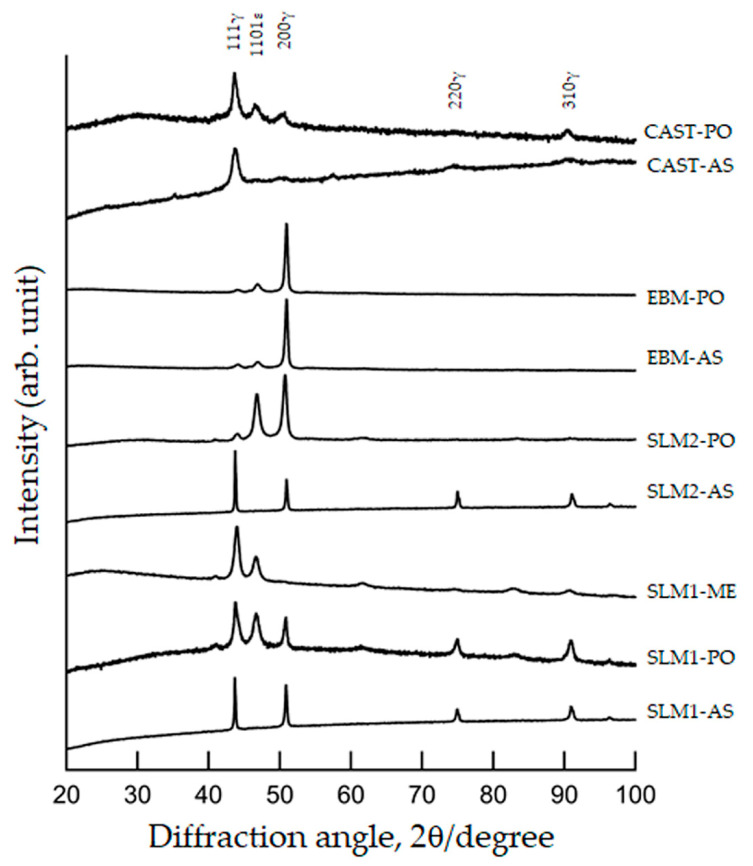
XRD patterns of SLM1, SLM2, EBM, and CAST specimens. See Section 2 in the main text for abbreviations.

Figure 1 shows the XRD patterns of the SLM1, SLM2, EBM, and CAST specimens. The peaks at approximately 43.6°, 50.7°, 74.5°, and 90.4° for the SLM1-AS and SLM2-AS specimens were assigned to the face-centered cubic *γ*-phase. The peaks at approximately 46.5°, 60.9°, and 82.5° in the other specimens were assigned to the hexagonal close-packed *ε*-phase. The *ε*-phase was observed in addition to the *γ*-phase in the SLM1-PO, SLM1-ME, SLM2-PO, and CAST-PO specimens, and the width at half maximum of the *γ*-phase peak increased, which did not happen in the as-built specimens. The (200)*_γ_* peak of the EBM-AS and EBM-PO specimens was strong, indicating that no change due to polishing was observed in the XRD patterns of the EBM specimen.

Table 2 lists the surface composition of the alloys analyzed using EPMA. Co, Cr, Mo, and W, which are the main constituent elements of each alloy shown in Table 1, were detected. The Co and Cr contents in the CAST-PO specimen were lower and higher than the value suggested in the manufacturer’s instructions in Table 1.

**Figure 2:**
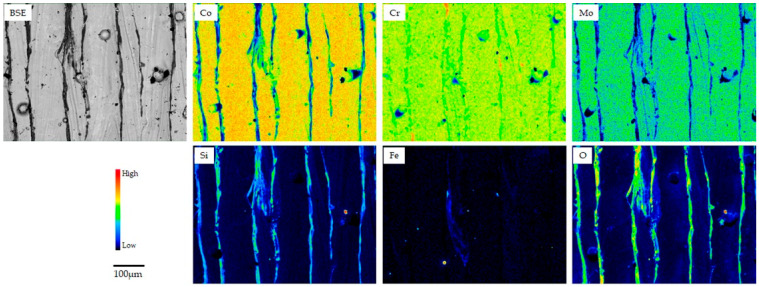
BSE and elemental mapping images of SLM1-AS specimen.

**Figure 3:**
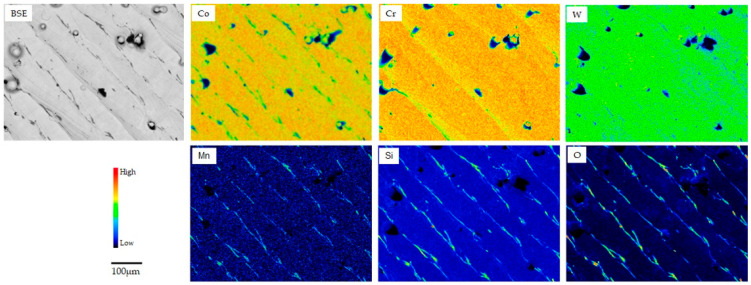
BSE and elemental mapping images of SLM2-AS specimen.

**Figure 4:**
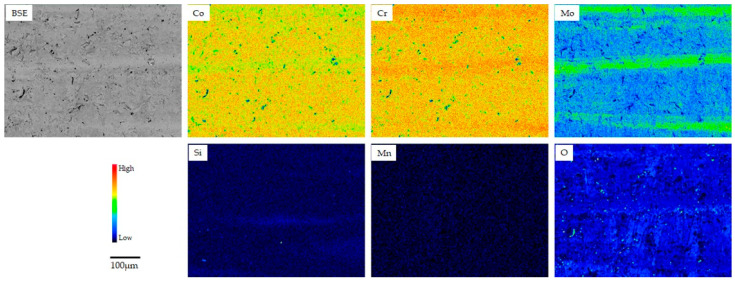
BSE and elemental mapping images of EBM-AS specimen.

**Figure 5:**
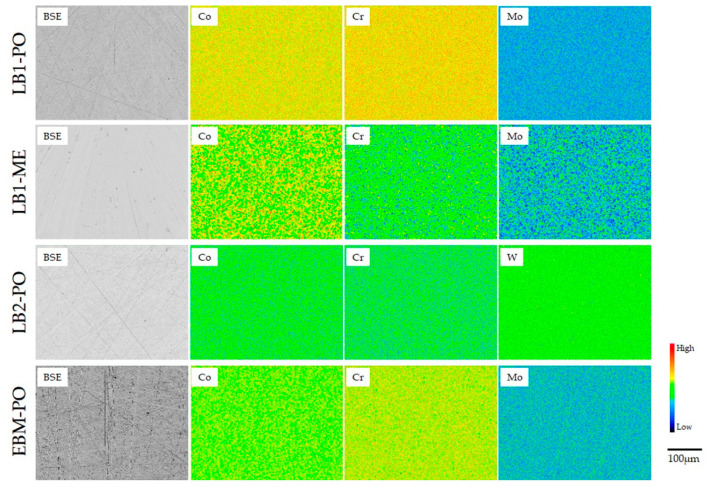
BSE and elemental mapping images of the SLM1-PO, SLM1-ME, SLM2-PO, and EBM-PO specimens.

**Figure 6:**
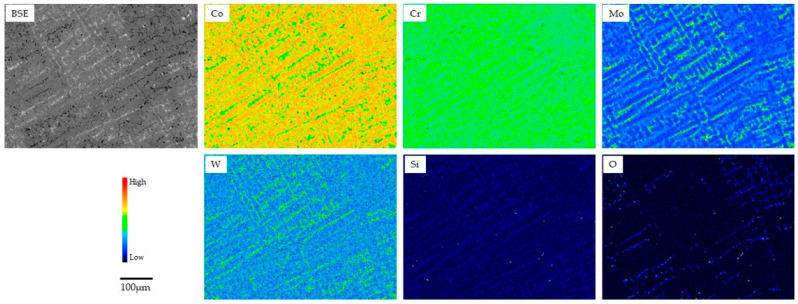
BSE and elemental images of the CAST-PO specimen.

Figure 2, Figure 3 and Figure 4 show the backscattering electron (BSE) and elemental mapping images of the constituent elements (Co, Cr, Mo, W, and/or Mn) and detected trace elements (Si, Fe, and O) in the SLM1-AS, SLM2-AS, and EBM-AS specimens, respectively. The BSE images represent the grain boundaries and sputter-like particles formed by laser scanning during the SLM1 and SLM2 processes. Large amounts of Si and O for the SLM1-AS specimen, and Mn for the SLM2-AS specimen, were observed at the boundaries. In addition, some Fe was observed in the SLM1-AS specimen. In the EBM-AS specimen, no sputter-like particles were observed; however, the images of Co, Cr, Mo, and Si showed a high-concentration area, and the elemental segregation was greater. In the SLM1-PO, SLM1-ME, SLM2-PO, and EBM-PO specimens, the elemental segregation observed in the AS specimens was eliminated by grinding and polishing (Figure 5). The BSE and elemental mapping images of the CAST-PO specimen show the dendritic segregation of Co, Cr, and Mo (Figure 6).

### 3.2. pH and Amount of Released Metallic Ions

**Figure 7:**
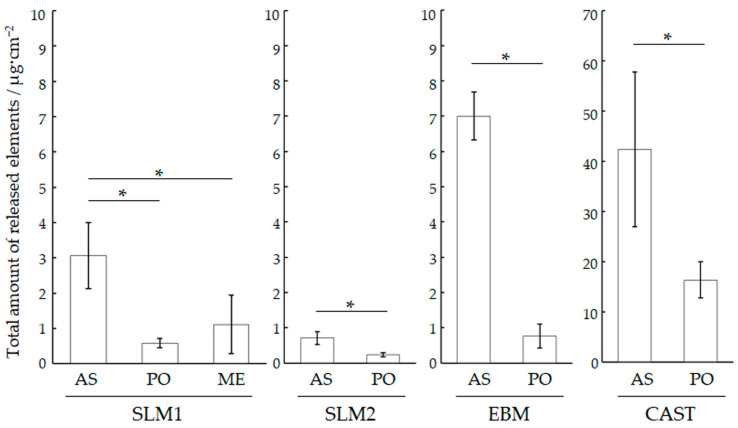
Total amount of metallic ions released from each specimen in an acidic saline solution for 7 days. * indicates the significant difference (*p* < 0.05). See “Materials and Methods” in the main text for abbreviations.

The pH values of the specimens after immersion are listed in Table 3. The acidic saline solution without the immersion of specimens (Blank) and the immersed solution were unchanged at pH 2.24 ± 0.03. Figure 7 shows the total concentration of the released metallic ions in the solution where each specimen was immersed. The CAST-AS specimen exhibits the highest number of metallic ions, followed by the CAST-PO, EBM-AS, SLM1-AS, and SLM2-AS specimens. The release of all AM specimens was less than 7 µg/cm^2^ after 7 days of immersion, whereas the CAST-AS specimen had the highest release at 42 µg/cm^2^. The total metallic ions released from SLM1-AS, SLM1-ME, and SLM1-PO were 3.1 μg/cm^2^, 1.1 μg/cm^2^, and 0.6 μg/cm^2^, respectively. The other PO specimens also showed a significant decrease in dissolution (*p* < 0.05). The total metallic ions released were below the ISO 22674: 2022 [25] requirement limit of 200 μg/cm² for all specimens.

Table 4 lists the concentration of each metal released from the PO specimens. The most released element from the SLM1 specimen was 0.2 μg/cm^2^ of Fe, which comprised approximately 0.75% of the composition of the SLM1 powder. The most abundant element, Co, was released from all specimens. Subsequently, Mo and Mn were released from the CAST specimens. The total amount of element released from the PO specimens created using AM was lower than that with casting (*p* < 0.05).

**Figure 8:**
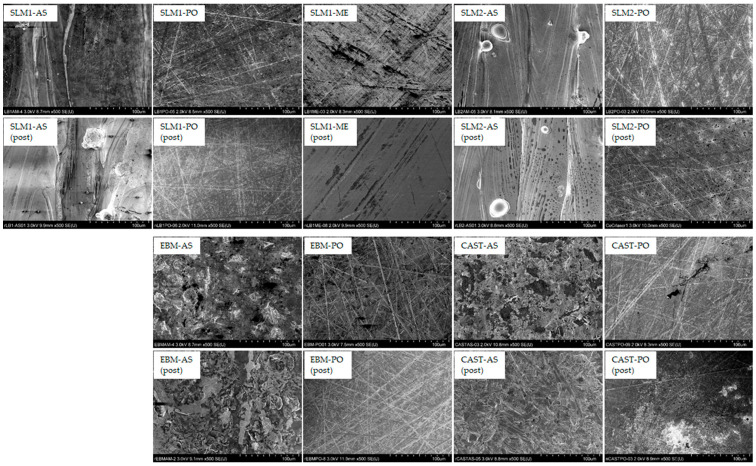
SEM images for SLM1, SLM2, EBM, and CAST specimens before and after immersion in an acidic saline solution for 7 days.

Figure 8 shows SEM images before and after immersion in an acidic saline solution. Corrosion morphologies were not clearly observed in the SLM1, SLM2, and EBM specimens. Conversely, only a small portion of CAST-PO specimens exhibited cast caves. No corrosion-like images were observed for any of the specimens after immersion.

## 4. Discussion

The use of metallic dental materials in the oral cavity is often associated with metal allergies [6,7,8,9,10]. In orthodontics, orthodontic wires, brackets, and bands are placed in the mouth for several months to years. Stainless steels and cobalt–chromium alloys, which exhibit excellent corrosion resistance owing to the formation of passive films on their surfaces, are used as materials in these devices [26]. However, the passive film may break down and corrode in the presence of chloride ions, causing ion release and the formation of corrosion products [27,28]. In the oral environment, saliva contains chloride ions, and the constituent elements of the alloy may be dissolved by corrosion. If the concentration of the released metallic ions exceeds the human threshold, allergic reactions may occur [9,10]. For crown restorations, metallic materials are generally fabricated by casting; however, the surface conditions are altered because of defects caused by casting and reactions with the investment material [14]. In this study, the release of metallic ions from cobalt–chromium alloys fabricated by AM was compared with that from cobalt–chromium alloys fabricated by conventional casting.

Binder jetting, material extrusion, directed energy deposition, and PBF are techniques that can be used in metal AM. In this study, PBF was performed by locally irradiating a layer of metallic powder with a laser or electron beam, which melted and solidified the metal powder. The metallic powder was then re-skimmed, and laser irradiation was repeated. The microstructure formed by this method differs depending on the modeling conditions (irradiation energy, energy density, laser scan speed, and building direction), and the mechanical properties and ductility of the material differ [13,14,15,16,17,29]. EBM is a method of melting and solidifying a single layer of powder locally, similar to SLM, with the difference being that the heat source for melting is an electron beam [30,31,32].

In the dental precision casting method, a pattern and mold are created, and the dental alloy is melted and cast to produce an individually fitted prosthetic device. Co-Cr alloys have a melting temperature of around 1400 °C, which is higher than that of dental gold alloys; therefore, special equipment, such as a high-frequency induction furnace, is required. In addition, a mold is required during casting, and casting defects may occur because of the reaction between the mold material and molten metal. When Co-Cr alloys are cast in a lump, casting shrinkage often causes a poor fit, and casting defects due to melting are also a problem.

Our study showed that specimens fabricated by casting (CAST) had the largest amounts of released elements, whereas their SLM and EBM counterparts had a lower release, indicating that the null hypothesis was rejected. The SEM observations revealed that the surfaces of the CAST specimens were blasted with alumina or glass beads to remove the surface investment material after casting. In this process, the surface becomes rougher, and the surface area increases. XRD patterns showed that the CAST specimen had an *ε* phase in addition to the *γ* phase in both AS and PO. EPMA analysis showed that the CAST specimen had a higher Cr concentration than the SLMs and EBM specimens; however, a cast cave was observed under SEM. Therefore, the increased surface area and cast cave in the CAST specimen may have reduced the corrosion resistance, resulting in ion release. 

A previous study reported that the amount of Co released in phosphate-buffered saline (PBS) from Co-Cr-Mo (CCM) alloys fabricated by SLM was less than 0.5 μg/cm^2^ for 7 days, and that lesser amounts of Cr and Mo were released [33]. When Co-Cr-W alloys were immersed in Hank’s solution, the highest amount of Co-release was less than 0.22 μg/cm^2^ for 7 days [34]. On the other hand, patients with metallic allergies often have allergic reactions to Co and Cr, but few patients develop such reactions [35]. The total ions released from the PO specimens in this study did not differ significantly from the literature. The majority of the released ions were cobalt, and a large amount of Fe was also found in SLM1. Release in the as-built and as-cast specimen was higher than that in the PO samples. Therefore, it is clear that the amount of release can be suppressed by grinding and polishing, although ion release was not completely zero even when fabricated by additive manufacturing. In this study, the XRD pattern for the SLM1 and SLM2 specimens showed that they were mainly composed of a *γ* phase. The *ε* phase, which is stable at low temperatures, was also observed in PO and ME specimens. The *ε* phase was also observed on the polished specimen surfaces by Takaichi et al. [36], which is consistent with our results. This is thought to be because the *γ* phase, which is stable at elevated temperatures, formed on the top surface of the specimen during laser irradiation, while the *ε* phase, which is stable at low temperatures, formed in its interior or during polishing and grinding. Compared to SLM, EBM is strongly oriented towards the face perpendicular to the building direction. Fabrication processes such as powder preheating and vacuuming may influence the orientation of the crystal. Therefore, the *γ* phase is predominant in EBM, and the (200) plane reflection of the *γ* phase was strong, indicating that the *γ* phase was highly oriented with respect to the building direction. Grain and/or melt–pool boundaries were present in the EBM and SLM specimens, and elements such as Mo in the EBM specimen were segregated at the boundaries. 

Released Fe from the SLM-AS specimen was the main metallic ion, and was present in the raw material; the amount of Co elution was 0.8 μg/cm^2^ for 7 days, and that of Cr and Mo was less than 0.2 μg/cm^2^. Spatter particles formed during laser irradiation were observed on the surface of the SLM specimens, in addition to the boundaries and melt pools. Considering the elemental distribution on the surface of the AS specimens fabricated by AM (AS), elemental segregation was observed at the fabrication boundaries, and Fe was locally distributed in SLM1. The iron compound dissolves easily in a saline solution. The acidic saline solution used in this study had a pH of 2.3, and the locally distributed Fe could react with and dissolve chloride ions.

More dissolution was observed in the CAST specimens than in the SLMs and EBM specimens in both AS and PO states. SEM images of the CAST specimens showed no difference before and after immersion. Holes that appear to be casting defects were observed in some areas (Figure 8). Casting defects and dendritic-like surfaces in the CAST specimen with the presence of carbides, in addition to the formation of a non-uniform passive film due to elemental segregation (mainly Cr segregation) [36,37,38,39,40,41], are involved in the corrosion reaction. Although the ratio of *γ* and ɛ phases did not change with polishing in EBM specimens, the ratio changed in SLM specimens and CAST specimens. The crystal structure of the Co-Cr alloys did not affect the release of metallic ions in the acidic saline solution. 

The polishing and grinding of the specimens further reduced the amounts of metallic ions released. Only lesser amounts of Co, Cr, and Mo were released from the SLM1-PO and SLM2-PO specimens, and the amounts released were comparable to previously reported values [33,34]. In SEM images taken before the immersion of SLM1, SLM2, and EBM specimens, it is clear that the polishing and grinding removed molten pools from the surface. As a result, the PO and ME surface became uniform, as shown in EPMA (Figure 5). Therefore, the presence of these molten pools caused the dissolution from specimens. The removal of one layer from the fabrication surface by grinding or polishing resulted in the removal of the boundaries and the segregation layer of the molten pool, and the surface became uniform. The *γ* and *ε* phases on the Co-Cr specimen showed a different crystal structure but similar composition, as shown in Figure 5. In other words, the removal of molten pools and/or sputter particles by polishing could create a uniform surface, resulting in no difference on the surface and possibly exhibiting superior corrosion resistance. This study suggests that Co-Cr alloys for dental appliances and prosthetic devices fabricated by additive manufacturing can meet the requirements of metallic ion release outlined in ISO 22674. On the other hand, the polishing and grinding performed during the fabrication of devices affect the metallic ion release. In future, the metallic ion release should be examined in the dental appliances and prosthetic devices themselves; in addition, the ion release in solutions containing other proteins and other substances in the oral cavity should be examined.

The SLM1-AS, SLM2-AS, and EBM-AS specimens in this study cannot be applied for clinical use in dentistry because AM fabrication limits the accuracy of fit in restorations [42]. However, the SLM1-AS, SLM2-AS, and EBM-AS specimens had lower metallic ion release than the CAST-PO specimens, and lower ion release than the standard release value outlined in ISO 22674:2022 (200 μg/cm^2^ for 7 days). Therefore, considering the corrosion resistance with respect to metallic ion release in the static immersion test, the released ion concentration can be used in the as-built specimens. When denture frameworks and clasps are used, polishing is required to improve the feeling on the tongue and reduce frictional resistance. Similarly, slippage between the orthodontic wire and bracket is an important consideration in the application of as-built brackets in AM. Therefore, polishing and mechanical grinding to reduce ion release are useful for improving the corrosion resistance. 

As a limitation of this study, cobalt–chromium alloys fabricated by SLM or EBM show better corrosion resistance in an acidic saline solution than that those fabricated by conventional casting in terms of metallic ion release. The amount of ion release can be reduced by removing one layer of the built surface via polishing or grinding to achieve a uniform surface composition.

Finally, Co-Cr alloys and titanium alloys are useful in removable partial dentures, owing to their excellent corrosion resistance, resulting from the formation of passive films. Although titanium alloys are known to be corroded by the fluoride in teeth, and vanish when used in the oral cavity [43], Co-Cr alloys have superior resistance to fluoride corrosion. In addition, Co-Cr alloys exhibit excellent wear resistance, which is important because the slots in orthodontic brackets must have a low coefficient of friction. Therefore, prosthetic devices and orthodontic brackets can be fabricated using additive manufacturing, and Co-Cr alloys could be useful because of their superior corrosion resistance in the oral environment. 

## 5. Conclusions

In this study, the corrosion resistance of Co-Cr alloys fabricated by SLM and EBM in PBF-AM was compared with the conventional casting technique in an acidic saline solution through the static immersion test according to ISO 10271: 2020. Within the limitations of this study, the following conclusions were drawn.

1. The segregation of the molten pool on the as-built SLM and EBM specimens fabricated using AM was observed, and the polished and machined specimens had homogenous surfaces.
2. The SLM and EBM specimens mainly had an *γ*-phase, but the polished and machined specimens showed an *ε*-phase.
3. Regardless of if polishing and machining were performed, the SLM and EBM specimens exhibited a lower metallic ion release than the CAST specimens. The total amount of metallic ions released from the SLMs and EBM specimens was less than 7 μg/cm^2^, but machining and polishing suppressed this amount.

These findings suggest that the fabrication of SLM and EBM using PBF-AM creates specimens with superior corrosion resistance in an acidic saline solution compared to conventional casting. The polishing and grinding on the as-built specimens can reduce ion release. Therefore, SLM and EBM in PBF-AM can be used for dental restorations and applications in clinical dentistry.

## Figures and Tables

**Table 1:** Nominal composition of powders and ingot for each specimen.

Specimen	Element (Mass%)				
	Co	Cr	Mo	W	Other
SLM1	Bal.	28.5	6.0	-	0.75 (Fe), ≤1.0 (Si, Ni, Mn), 0.75 (C)
SLM2	60.5	28.0	-	9.0	1.5 (Si), (Mn, N, Nb)
EBM	Bal.	28.0	6.0	-	0.15(N), ≤1.0(Mn)
CAST	59.5	29.2	6.6	2.6	2.1 (Si, Mn, N, C)

**Table 2:** The chemical composition of each specimen with or without polished/mechanical ground, determined by EPMA (mass%).

Specimen		Element (Mass%)					
		Co	Cr	Mo	W	Si	Other
SLM1	AS	63.4	29.7	5.4		0.7	Fe, Mn
	PO	63.4	30.0	5.7		0.7	Fe
	ME	62.7	30.4	5.6		0.9	Fe
SLM2	AS	61.2	28.5	-	8.8	1.5	
	PO	60.8	29.5	-	8.4	1.3	
EBM	AS	66.2	28.4	4.9		0.6	Mn, Ni
	PO	65.4	28.4	4.8		0.6	
CAST	AS	61.2	29.3	5.6	2.8	0.8	Al
	PO	54.5	33.1	7.4	4.0	0.9	

**Table 3:** The pH of the acidic saline solution in the acidic saline solution after the immersion of each specimen.

	SLM1	SLM2	EBM	CAST
AS	2.26 ± 0.04	2.24 ± 0.00	2.21 ± 0.01	2.25 ± 0.01
PO	2.26 ± 0.04	2.25 ± 0.01	2.21 ± 0.01	2.22 ± 0.01
ME	2.26 ± 0.03			

Blank: 2.24 ± 0.00.

**Table 4:** Metallic ion concentrations released from each PO specimen in an acid saline solution during a 7-day immersion.

Specimen	Element (μg/cm^2^)							Total
	Co	Cr	Mo	W	Mn	Fe	Ni	(μg/cm^2^)
SLM1	0.22	0.05	-	-	0.01	0.22	0.08	0.58 ± 0.13 ^a^
SLM2	0.21	0.02	-	-	-	-	-	0.24 ± 0.06 ^a^
EBM	0.74	0.05	0.03	-	0.01	-	-	0.76 ± 0.34 ^a^
CAST	12.52	0.62	1.89	0.61	0.58	-	-	16.35 ± 3.60 ^b^

Welch’s one-way ANOVA followed by Tukey’s test. Different lowercase letters in the total amount of released ions are significantly different (*p* < 0.05). See “Materials and Methods” in the main text for abbreviations.

## Data Availability

The original contributions presented in the study are included in the article, further inquiries can be directed to the corresponding author.

## Abbreviations

AM	Additive manufacturing
AS	As-built
BSE	Backscattering electron
CAD	Computer-aided design
CAM	Computer-aided manufacturing
Co-Cr	Cobalt–chromium
EBM	Electron beam melting
EPMA	Electron probe microanalyzer
SEM	Scanning electron microscope
SLM	Selective laser melting
PBF	Powder bed fusion
ICP	Inductively coupled plasma optical emission spectrometer
XRD	X-ray diffractometer
